# From Income to Capital Breeding: When Diversified Strategies Sustain Species Coexistence

**DOI:** 10.1371/journal.pone.0076086

**Published:** 2013-09-27

**Authors:** Pierre-François Pélisson, Marie-Claude Bel-Venner, David Giron, Frédéric Menu, Samuel Venner

**Affiliations:** 1 Université de Lyon, Lyon, Université Lyon 1, CNRS, UMR5558, Laboratoire de Biométrie et Biologie Evolutive, Villeurbanne, France; 2 Biological Control and Spatial Ecology Laboratory (LUBIES), Université Libre de Bruxelles, Brussels, Belgium; 3 Institut de Recherche sur la Biologie de l’Insecte, UMR 7261 CNRS - Université François Rabelais, Parc Grandmont, Tours, France; French National Institute for Agricultural Research (INRA), France

## Abstract

There is empirical evidence of many diversified ways for energy to be acquired and allocated to reproduction, notably with strategies ranging from strict income breeding (females fueling their gametes with energy gained concomitantly during reproduction) to strict capital breeding (females storing nutrients prior reproduction). Until now, the question of whether diversification of these strategies might impact the way communities are organized has not been considered. Here, we suggest that diversified resource allocation strategies among competing species may contribute to their coexistence. We examined this hypothesis by focusing on communities composed of four phytophagous insect species that coexist and compete for egg-laying sites. From wild-caught females, we determined precisely the breeding period of each species and we described their resource acquisition and allocation to reproduction dynamics. We quantified in each species the total amount of larval energy stored by newly-emerging females and then monitored the total energy budget of females caught in the field before and throughout their breeding period. We found that the four sibling weevil species are markedly segregated along the income-capital-breeding continuum, which is correlated with clear time partitioning in their laying activity. Our results suggest that diversified resource allocation strategies might contribute to time partitioning of plant resources exploitation and thus indirectly to their coexistence. This work should further encourage studies examining the extent to which competitive coexistence might be affected by diversification of income-capital breeding strategies together with the intensity of interspecific competition, and considering the divergent evolution of these strategies.

## Introduction

Acquiring energy from limited resources and allocating it to various physiological pathways is a central issue for all organisms (e.g. [1-3]). The corresponding strategies of energy Acquisition and Allocation to Reproduction (AAR) have been empirically shown to vary considerably across species within the animal and vegetal kingdoms (see 4 for a review). These strategies can be pinpointed along a continuum extending from strict capital breeding, with females storing nutrients prior breeding *sensus* Jervis [5], up to strict income breeding, where oogenesis is fueled by energy gained concomitantly [5–11].

AAR strategies have been explored at various organization levels, from underlying molecular processes (*eg.* [12]) to population dynamic consequences (see 4 for a synthesis). For example, they are involved in animal behaviors [8] and they were shown to be correlated with the eruptive and cyclic population dynamics among Lepidoptera [13]. Yet, their implications at the community level have received much less attention so far. In a given environment, an optimal pattern of resource acquisition and allocation to reproduction is often expected [4]; contrary to this view, we suggest that species competing for a single resource may diversify their AAR strategies along an income-capital gradient in a way that favor niche-partitioning and thus their coexistence. Due to the lack of empirical data available to explore this hypothesis, we identified whether sibling species competing with each other for the same resource occupied contrasted positions along the income-capital breeding continuum and whether this could be correlated to their reproduction period partitioning.

We focused on insect communities composed of four weevil species - *Curculio glandium* (Marsham), *C* *. elephas* (Gyllenhal), *C* *. pellitus* (Boheman), and *C* *. venosus* (Gravenhorst) (Coleoptera, *Curculionidae*), that are specialized on oak trees ( *Quercus* spp.). These species coexist on the same individual host plants [14-17], where they compete for the use of oak acorns as their unique egg-laying sites [18]. From early June to the end of summer, weevil females deposit eggs into oak acorns where larvae complete their development before burrowing into the soil and entering in winter diapause. Because oak trees are known to be “mast-seed” species (i.e., they produce seeds massively but intermittently from one year to another [19]), the number of egg-laying sites available for the four weevil species varies considerably and, to some extent, unpredictably across years. In addition, the resource availability also varies during the breeding season of insects due to the well-described massive abortion of both flowers and undeveloped fruits occurring in mid-summer (e.g. [20-22]).

In such unpredictable and competitive environment, stable coexistence of the four weevil species studied seems to be ensured by a temporal partitioning of the plant resource use. This is allowed by marked differences observed both in insect dormancy capacities and their laying period during the reproductive period [18]. Within-year partitioning is generally expected to favor species coexistence since differences in the timing of resource consumption is one requirement of the storage effect [23,24]. More specifically, in the studied system, because the limiting resource (oak acorns) fluctuates both across and within years, time partitioning of the resource use during the insect reproduction period is expected to significantly impact the community structure [18]. On one hand, early-laying species should be advantaged compared with late-laying species (especially when competition is severe) because they have prior access to oak acorns. On the other hand, due to their ability to lay eggs in already mature and unparasitized acorns, species laying eggs later in the season might exploit fruits more efficiently than early-emerging species. Species laying late in the season would therefore have an advantage over early-laying species under weak competition for acorns (e.g., years with large seed crop and low density of breeding adults). Accordingly, variations in plant resource availability induce various levels of competition that should favor consecutively each insect species due to their oviposition time partitioning [23]. In this study, we suggest that such partitioning of resource use during the insect reproductive season is linked to distinct AAR strategies between the four insect species, which would be characterized by different positions along the income-capital breeding continuum.

In a previous study, two main types of energy strategies have been identified amongst the oak weevil species studied based on whether newly-emerging females had mature oocytes and on whether weevils consumed food at adulthood [25]. Three species – C*. glandium,* *C* *. pellitus* and *C* *. venosus* – were shown to be fully synovigenic (i.e., females emerging without or very few mature eggs [26]) and were likely to be income breeders *sensu stricto* [27] as their gamete production relied mostly on nutrients acquired during their adult life [25]. In contrast, the fourth species – *C* *. elephas* – was found to be proovigenic (i.e. the lifetime egg production was already available upon adult emergence) and capital breeder, since wild-caught females had mature oocytes at emergence, and did not feed during adulthood [25].

Because of its qualitative nature, this dichotomous description failed to distinguish among three of the four species as it was not designed to precisely identify their position along the capital-income breeding continuum. To detect a possible diversification of energy strategies displayed by these competing species, we developed a finely-tuned quantitative field study to monitor their lifetime energy dynamics in these four species. Accordingly, for each species we measured the total amount of energy in female at emergence and in females caught later in the field throughout their adult life. We determined for each species the egg-laying period and the lifetime kinetics of reproductive investment of females to finely connect the within-year partitioning of plant resource use observed between the four species to the strategies of energy acquisition and allocation to reproduction.

## Materials and Methods

Female insects belonging to the four weevil species were surveyed and sampled in two independent communities, each of these being located on an isolated oak tree: the two ~150-200 year-old trees are 30 km apart from each other in a fragmented, agricultural landscape near Lyon (France) (site A ( *Quercus* *robur* ): N45° 35′; E5° 01′; site B ( *Q* *. petraea* ): N45° 45′; E5° 16′; see 18 for details).

### Ethics statements

This study was carried out on private lands, and we confirm that the landowners gave us permission to conduct this study. Furthermore, we confirm that no specific permission was required for this field study since it did not involve any endangered or protected species.

### Insect sampling

For each weevil species we captured “newly-emerged” (see below) females at sites A and B before they could feed as adults. To obtain such females, we initially harvested mature acorns that had dropped onto a net placed on the ground at the base of each of the two trees and covering half the surface of their vegetal cover. The fruits were counted and then placed in wire-netting boxes in an outdoor arena that allowed us to collect and count all the mature weevil larvae that freely extracted from the acorns. Mature larvae were collected on the day they left the fruit, which occurs yearly from September to December. Each of these larvae was randomly assigned to one of several covered, water-permeable plastic receptacles that had previously been filled with sifted soil and buried under each host tree. This operation was repeated during four consecutive years (from 2005 to 2008), with larvae being assigned each year to new, virgin receptacles in order to know the number of years each larva spent underground (from one to three years, depending on the species [18]). Each receptacle therefore allowed larvae from a known cohort and tree to pursue its development in confined, semi-natural conditions until the adults emerged. In 2009 we surveyed adult emergence in 46 such receptacles on a weekly basis from early March to end of September. As soon as the first adult emerged in any of the receptacles, we started a daily survey and collected all adults until none emerged in any of the devices for at least eight consecutive days. For the purpose of this study, we collected 61 females within three hours after emergence (12 *C* *. venosus* (8 and 4 at the sites A and B, respectively), 13 *C* *. pellitus* (7 and 6), 20 *C* *. glandium* (11 and 9), and 16 *C* *. elephas* (8 and 8)).

The same year, we also “live-trapped” weevil females that were naturally present on oak trees, regularly throughout the breeding season. Since for a given species, adult emergence occurs synchronously within a few days, females sampled early in the season are younger than those ones sampled at the end of the breeding period [25]. This sampling was conducted weekly in the two insect communities from adult emergence (April) until no adult was found on either tree at two consecutive sampling sessions (mid-October). To ensure a consistent sampling effort throughout the sessions, 6 and 7 branches were randomly selected on trees A and B, respectively, and were consistently shaken with the same number of beats early in the day (see detailed method in [18]). At each tree, two females per weevil species and per sampling session were randomly selected to be included in our analyses: this ensured a balanced sampling design throughout the season (i.e., a total of 197 females including 43 *C* *. venosus* (21 and 22 at the sites A and B, respectively), 40 *C* *. pellitus* (27 and 13), 88 *C* *. glandium* (46 and 42), and 26 *C* *. elephas* (11 and 15)).

The sex and the species of newly-emerged as well as of live-trapped weevils were identified using morphological criteria [14]. Each selected weevil was then brought back to the laboratory in a cold box, measured for its body length to the nearest 0.1 mm with a binocular microscope (Zeiss stemi-C; Zeiss, Illkirch, France; magnification x16), weighed to the nearest 0.1 mg (balance: Scaltec SBA 32) and individually frozen dry at -20°C.

### Determination of the egg-laying period

To analyze the lifetime dynamics of female fecundity, the newly-emerged as well as the live-trapped females were dissected under a binocular microscope and their total oocytes were counted (mature and immature). To detect the onset of the breeding period, we also checked for the presence of sperm in females’ spermathecae using DAPI coloration (Di Aminido Phenyl lndol). For each spermatheca, we used a 20μl staining solution (15μl of Vectashield without DAPI blended with 5μl of Vectashield with DAPI) to fix the coverslip on the glass slide. Following staining, each sample was stored two days at 4°C in the dark before checking the presence of sperm with a fluorescence microscope (Imager Z1 AxioCam).

### Determination of the global energy budget

We assessed the total amount of energy stored by weevil females sampled throughout the breeding season. For that purpose, we conducted colorimetric analyses to simultaneously assay on the same individuals the total lipid, total soluble carbohydrate and total soluble protein contents (see detailed method in [26]). The total energy budget of a female was then computed using the following equivalence: 16 Joules per mg carbohydrates or proteins and 37.65 Joules per mg lipids [27,28]. Each value was divided by the female’s body length to account for differences in body size existing among and within the four weevil species (Kruskal-Wallis χ^2^_df=3_ = 117, *P< 0.0001*; body length of *C* *. venosus* females (n=55): 5.81± 0.36 mm (mean±se); *C* *. pellitus* (n=53): 6.17 ± 0.45 mm, *C* *. glandium* (n=108): 5.24 ± 0.50 mm; *C* *. elephas* (n= 42): 5.97; ± 0.43 mm). All subsequent analyses are made using the amount of energy corrected for body size.

### Data analysis

We tested in each of the four weevil species whether the energetic status of the females varied from their emergence above ground (“newly emerged” females) to the time they were actively ovipositing on trees (“live-trapped” females). We built up a linear model using a contrast procedure to test whether the total amount of energy stored in the female’s whole body differed, on average, between these two periods.

To check for possible time partitioning in the egg-laying period among the four species, we used a logistic regression (generalized linear model with a binomial distribution of error) to test whether the interaction between the species and the sampling date affected the probability that a female mated and matured oocytes.

In each species, the seasonal dynamics of the total amount of energy (together with that of the specific nutrients taken separately, i.e., total lipids, carbohydrate and proteins; see Figure S3) stored by females was analyzed using a linear model including three explanatory variables: the capture day (1-Time), and its corresponding squared and cubic values (2-Time² and 3-Time^3^, respectively) that accounted for possible non-linear time variations of the amount of energy or specific nutrients levels. For each species, the intercept was set up as the average amount of the total energy (the nutrient category) stored by females on the average emergence day.

We tested whether the four weevil species had distinct egg-maturation dynamics across time using a generalized linear model with a Poisson distribution of errors that predicted the number of mature oocytes produced by a female as a function of its species and of its sampling date. All analyses and figures were performed with the R freeware statistical environment v 12 (R Development Core Team (2011); http://cran.at.r-project.org).

## Results

**Figure 1:**
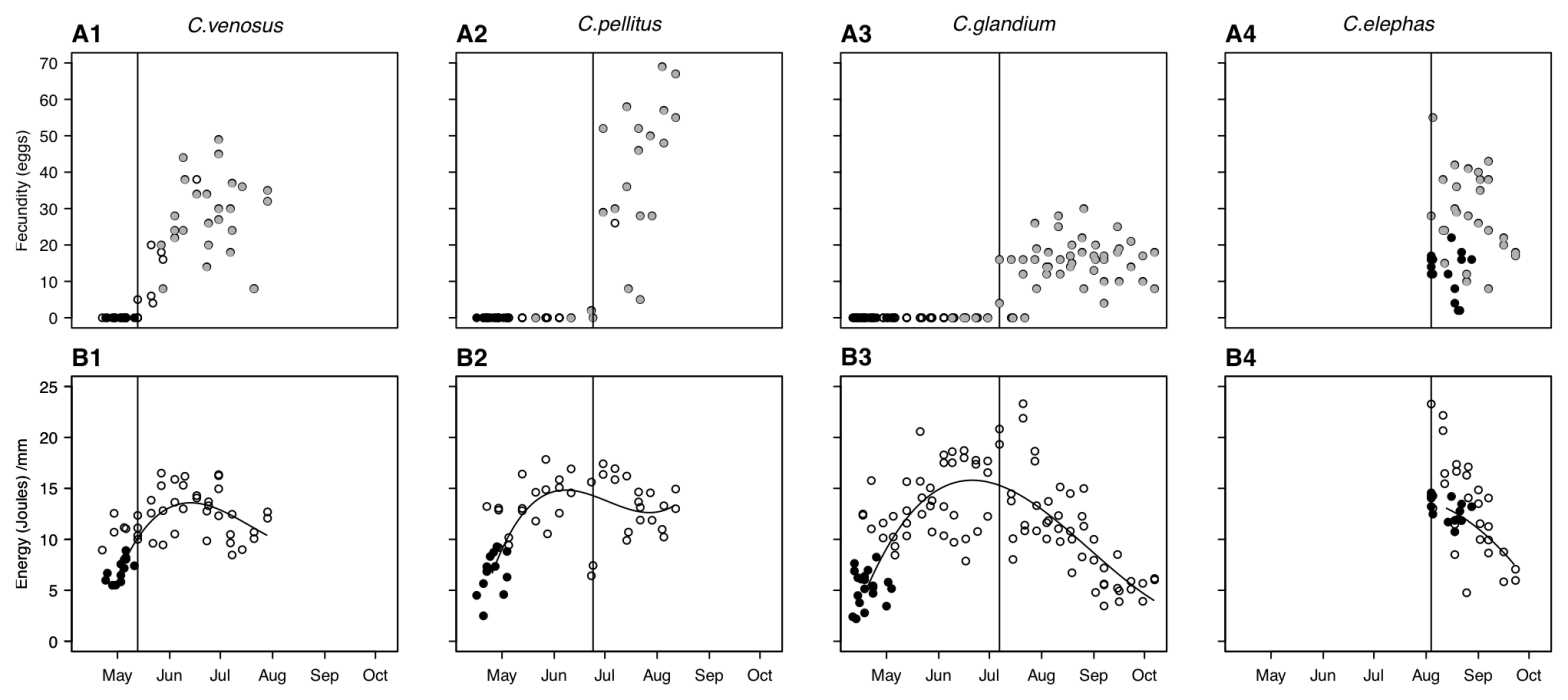
Seasonal dynamic of the whole-body energy budget of wild-caught females belonging to the four *Curculio* species. Upper row (A): Number of oocytes contained in the females ovaries. Mated and virgin females are distinguished according to the occurrence of sperm in their spermathecae and are individually represented with full grey and black circles, respectively. Lower row (B) shows total energy budget of female insects. Newly-emerged (full black circles) and lived-trapped females (open circles) are shown. For each species, the vertical line represents the starting date of oogenesis (C. *venosus* 05/13/2009 -m/d/y- ; *C*. *pellitus* 06/23/2009; *C*. *glandium* 07/07/2009; *C*. *elephas* 08/05/2009).

**Figure 2:**
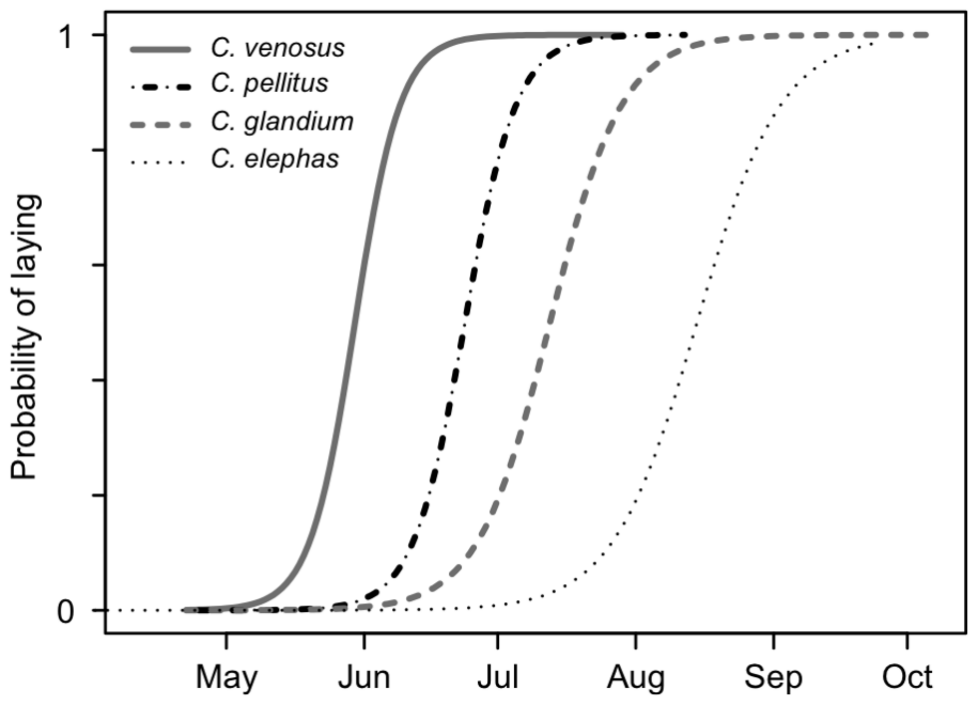
Seasonal time partitioning among the four competing species. The logistic regression models of laying probabilities over time significantly differ among the four species. Only mated females with mature oocytes are considered as being ready to lay (probability=1). Virgin and/or immature females (i.e., with no ovarian development) are considered to have a null probability of laying.

Upon emergence, females of the four species overall differed in their total energy budget (F_3,57_= 83.34 *P* < 0.0001; n_*C.venosus*_= 12, n_*C.pellitus*_= 13, n_*C.glandium*_=20, n_*C.elephas*_= 16; see Figure 1 B1-B4, Figure S1) as well as in the number of their mature oocytes: *C* *. elephas* females were the only ones to have mature eggs at emergence (Figure 1; panels A1-A4) and compared with the three other species, they stored the greatest amount of teneral reserves at adult emergence (contrast procedure analysis: t_57_=15; *P*<0.0001). The energy stored by newly-emerged individuals compared to ovipositing females also led to distinct conclusions across the four species (interaction between the species and the mating status: F_3,249_=8.6, *P < 0.0001*; n_*C.venosus*_ = 55, n_*C.pellitus*_ = 53, n_*C.glandium*_ =108, n_*C.elephas*_ = 42). Indeed, the three synovigenic species ( *C* *. glandium* , *C* *. pellitus* and *C* *. venosus* ) all experienced a net increase in their total energy budget in the first part of the breeding season, thereby providing evidence of food intake by these insects in the field. We detected a second phase following mating, with total energy decreasing simultaneously to increasing effort toward egg production (Figure 1; panels 1-3). Although similar patterns of seasonal dynamic of energy acquisition were found among the three species, between-species differences occurred in the time that elapsed between female emergence and initiation of oogenesis, which provides the species with distinct positions along the capital-income breeding continuum. Hence, *C* *. venosus* females exhibited an income breeder-like strategy since they first showed rapid net energy gain and then soon started maturing eggs while still accumulating energy (Figure 1; panels A1, B1). By contrast, *C* *. pellitus* as well as *C* *. glandium* females first gained energy at a slow rate and over a long period of time before showing a net decrease concomitant with mating and egg maturation. These two synovigenic species are therefore considered to be capital breeders, even if resources invested in egg production is mainly acquired at adulthood (a strategy that could be called ‘adult capital breeder’). Furthermore, we detected subtle differences between *C* *. pellitus* and *C* *. glandium* since females, while emerging at the same time, did not initiate oogenesis synchronously and were unequally long-lived, which resulted in distinct egg-laying periods. We thus found that the four species overall exhibited clearly distinct patterns of temporal dynamic of their ovarian development (χ^2^_df=3_ = 288, *P*<0.0001) and distinct egg-laying periods (logistic regression: χ^2^_df=3_ = 253, *P*<0.0001; Figure 2; Figure S2; table S1).

*C* *. elephas* also markedly differed from the three other species as the total energy stored by newly-emerged females did not differ, on average, from breeding females (F_1,40_^≈^ 0; *P* =0.98; Figure S1; panel D). Females were found capitalizing most of their energy at the larval stage even though some of them (about 30%) also acquired energy at the adult stage (see Figure 1 panel B4). However, food intake at adulthood does not seem to be required for ovarian development since all *C* *. elephas* females readily mated and matured eggs upon emergence, and experienced continuous decline in their total reserves thereafter (Table 1; Figure 1 panels B1-B4).

**Table 1:** Seasonal dynamic of the amount of energy stored by females of each species in their whole body.

***Species***		***Df***	***F value***	***P (>F)***	***Significance***
*** C. venosus ***	Time	1;40	116.09	<0.0001	***
	Time2	1;40	34.87	<0.0001	***
	Time3	1;40	1.46	0.23	NS
*** C. pellitus ***	Time	1;37	128.79	<0.0001	***
	Time2	1;37	31.19	<0.0001	***
	Time3	1;37	6.39	0.0158	*
*** C. glandium ***	Time	1;85	177.31	<0.0001	***
	Time2	1;85	190.38	<0.0001	***
	Time3	1;85	10.76	0.0015	**
*** C. elephas ***	Time	1;23	9.13	0.0060	**
	Time2	1;23	0.14	0.70	NS
	Time3	1;23	2.99	0.09	NS

NS:P>0.05 (non significant), * *P* < 0.05; ** *P* < 0.01; *** *P* < 0.001.

We used a linear model to test whether time of the year (and it square and cubic value) influences the amount of energy contained in the females (corrected for body length).

In every species, all nutrient categories taken separately experienced the same seasonal dynamics as the total energy budget except carbohydrate in *C* *. elephas* females that monotonously increased during the breeding season (See Figure S3 panel C4).

## Discussion

A single strategy of resource acquisition and allocation toward reproduction is classically predicted to evolve in a given environment as an optimal response to the environmental availability and/or predictability of the resource (e.g., [4,9,29]). In this field study, we described the lifetime energy budget of four weevil species that belong to the same communities and compete for the same and fluctuating egg-laying sites (oak acorns). We monitored wild-caught females for the seasonal dynamics of their nutrient reserves in connection with their ovarian development. Contrary to what is classically expected, we found evidence that four weevil sibling species belonging to the same communities differentially acquired energy and allocated it toward breeding. As a consequence these four species could be clearly discriminated along an income-capital breeder axis, which could be linked with the seasonal partitioning of the plant resource use during the insect reproductive season.

*C* *. venosus* appears to be the most extreme income breeder of the four species, since young females started investing energy early in the breeding season and thus, laid eggs concurrently with energy gain (Figure 1, Figure S2). This strategy allows *C* *. venosus* being the first of the three early-emerging species [25] to mature and lay eggs (Figure 1, panel A1; Figure 2; Figure S2). This ensures females prior access to the resource and may enable them to find sufficient acorns locally even on years when the resource is scarce. However, since *C* *. venosus* eggs are laid before flower abortion in July [22,30], this may entails fitness costs either due to the elevated risk of larval development failure if females lay their eggs indiscriminately into acorns that may later abort, or due to the waste of time and energy that they would spend discriminating and selecting safe –fertilized- oak acorns for egg laying.

Analyzing the seasonal dynamics of their energy budget reveals that *C* *. pellitus* and *C* *. glandium* females are both capital breeders since adult females first stored energy before starting maturing eggs (Figure 1; panels B2, B3). Without accounting for this seasonal dynamic, these two species would have erroneously been assigned to income breeders owing to the adult origin of the energy required for breeding [25]. None of these two species have matured eggs upon emergence in early spring (Figure 1 [25]), before any oak acorn can be fertilized [31]. The time *C* *. pellitus* and *C* *. glandium* adult females spent feeding postponed egg laying after flower abortion has occurred, thereby reducing the risk of laying in a fruit unsuitable for larval development. We further showed that these two species exhibited time partitioning in their breeding activity within the season (Figure 2): *C* *. pellitus* females acquired energy and allocated it to reproduction faster than *C* *. glandium* , and were thus able to use oak acorns earlier in the season. Furthermore, mainly due to greater lifespan, *C* *. glandium* females laid eggs during a larger timeframe than *C* *. pellitus* , while still maintaining a lead over the latest species, *C* *. elephas* .

Lastly, we found that *C* *. elephas* females had mature oocytes as soon as they emerged in August and capitalized energy of exclusively larval origin before starting breeding (Figure 1, panels A4, B4): this confirms a previous study suggesting that *C* *. elephas* females were capital breeders [25]. However, we were able to detect few females that experienced a net energy gain early in the breeding season (Figure 1, panels B4, Figure S3). Yet, even if food intake occurs during adulthood, the nutrients ingested might not necessarily be allocated to reproduction [25] and further work is needed to understand the issue of such food intake by a minority of adults in this species. *C* *. elephas* is nevertheless the latest weevil species exploiting oak acorns in the breeding season. This strategy would be adaptive whenever the breeding efficiency of females on mast-seed years (i.e., when egg-laying sites are highly abundant locally) outweigh their inability to reallocate energy in flexible manner (e.g., toward either breeding or adult maintenance) the years with low fruit availability.

Our result suggest that the contrasted AAR strategies observed might underlie time partitioning in the breeding activity and thus, would most likely contribute to sustain the storage effect in communities of insect species competing for oak acorns, which effect is expected to promote their coexistence [13,23,24]. Alternately, it cannot be ruled out that interspecific competition would have directly promoted temporal niche partitioning that, in turn, would have favoured diversification of traits, among which AAR strategies, in a way that maximizes the lifetime reproductive success of insects in their specific breeding period. While it is too early to conclude which of the two mechanisms is the right one, our correlative study is the first to consider the possibility for AAR strategies diversification to be involved in the coexistence of competing species and to gather empirical data compatible with this hypothesis. Indeed, numerous empirical studies made on plants, vertebrates or insects have reported a large array of strategies along a continuum extending from pure income to pure capital breeding [5,8,9,11,32]. Positioning of a species along this continuum is usually considered to depend on the internal status of targeted organisms, on the nutrient type considered, and on the degree of predictability of the resource in the environment [4,11,33,34]. In parallel, several studies disconnected with the community ecology literature reported different AAR strategies developed by species at the same trophic level and living sympatrically such as plants (e.g. [35]) or animals (birds (*e.g* [36]), mammals (*e.g* [37,38]) and insects (*e.g* [27,39]). Intriguingly, none of these studies investigated whether these species actually competed with each other or considered the possible impact of their strategies on community assembly. The extent to which AAR strategies are diversified would greatly depend not only on environmental variability, but also on the competition strength between the coexisting species. Unlike the classical theory that predicts a unique, optimal strategy in a given environment, we suggest that disturbed environments may promote the diversification of AAR strategies among competing species which in turn may favor their stable coexistence. Our work should therefore encourage further studies providing empirical evidence of the impact of income-capital breeding strategies at the community level.

## Supporting Information

Figure S1 **Total energy budget of the four Curculio species.** Total amount of energy stored in newly-emerged (grey bars) and lived-trapped breeding (white bars) females of the four Curculio species. Box-plots: horizontal bold line, median; box, lower and upper quartiles; dashed lines, 95% confidence interval.
(DOCX)

Figure S2 **Time partitioning among the four competing species.** Representation (in dark) of logistic regression (logit link assuming a binomial distribution of errors) of the ovarian dynamics for each species (presence or absence of eggs in ovaries). The grey lines represent the seasonal dynamic of the whole-body energy budget of females belonging to the four *Curculio* species, presented in the Figure 1 and table 1. The comparison of the two models shows the acquisition and allocation dynamics in reproduction.
(DOCX)

Figure S3 **Seasonal nutrient dynamic in females of the four *Curculio* species.** Top (line A) Seasonal dynamic of total lipids for the four species (*C. venosus* A1; *C. pellitus* A2; *C. glandium* A3; *C. elephas* A4). Medium (Line B) Amount of total proteins Low (Line C): Amount of total carbohydrates. Data shown for newly-emerged females (full circles) and lived-trapped females (open circles).
(DOCX)

Table S1 **Analyses of the time partitioning between species in the breeding season.** Logistic regressions of laying probabilities differ between species (interactions species: date). All pairwise tests are significant even by applying a Bonferroni correction for multiple tests.
(DOCX)

## References

[1] Van Noordwijk A, De Jong G (1986) Acquisition and allocation of resources: their influence on variation in life history tactics. Am Nat 128: 137–142. doi:10.1086/284547.

[2] Karasov WH (1986) Energetics, physiology and vertebrate ecology. Trends Ecol Evol 1: 101-104. doi:10.1016/0169-5347(86)90034-0. PubMed: 21227790. 21227790 10.1016/0169-5347(86)90034-0

[3] Boggs CL (1992) Resource Allocation: Exploring Connections between Foraging and Life History. Funct Ecol 6: 508-518. doi:10.2307/2390047.

[4] Stephens PA, Boyd IL, McNamara JM, Houston AI (2009) Capital breeding and income breeding: their meaning, measurement, and worth. Ecology 90: 2057-2067. doi:10.1890/08-1369.1. PubMed: 19739368. 19739368 10.1890/08-1369.1

[5] Jönsson IK (1997) Capital and income breeding as alternative tactics of resource use in reproduction. Oikos 78: 57-66. doi:10.2307/3545800.

[6] Stearns SC (1992) The evolution of life histories. In: Krebs JR Davies NB Oxford University Press UK pp. 249.

[7] Bonnet X, Bradshaw D, Shine R (1998) Capital versus income breeding: an ectothermic perspective. Oikos 83: 333–342. doi:10.2307/3546846.

[8] Meijer T, Drent R (1999) Re-examination of the capital and income dichotomy in breeding birds. Ibis 141: 399-414.

[9] Houston AI, Stephens PA, Boyd IL, Harding KC, McNamara JM (2006) Capital or income breeding? A theoretical model of female reproductive strategies. Behav Ecol 18: 241-250. doi:10.1093/beheco/arl080.

[10] Johnson RA (2006) Capital and income breeding and the evolution of colony founding strategies in ants. Insect. Soc. 53: 316-322.

[11] Warner DA, Bonnet X, Hobson KA, Shine R (2008) Lizards combine stored energy and recently acquired nutrients flexibly to fuel reproduction. J Anim Ecol 77: 1242–1249. doi:10.1111/j.1365-2656.2008.01442.x. PubMed: 18637855. 18637855 10.1111/j.1365-2656.2008.01442.x

[12] Arrese EL, Soulages JL (2010) Insect fat body: Energy, metabolism, and regulation. Annu Rev Entomol 87: 207-225. PubMed: 19725772. 10.1146/annurev-ento-112408-085356 PMC3075550 19725772

[13] Tammaru T, Haukioja E (1996) Capital breeders and income breeders among Lepidoptera - Consequences to population dynamics. Oikos 77: 561-564. doi:10.2307/3545946.

[14] Hoffmann A (1954) Faune de France. Coleopteres Curculionides, (Deuxième partie). Fédération Française des Sociétés de Sciences Naturelles, Paris, France.

[15] Coutin R (1992) Original characteristics of the evolving cycles of some European weevil species: Curculio elephas Gyll., C. nucum L., C. glandium Marsh., C. venosus Grav. and C. villosus F. Memoires de la Societe Royale Belge d’Entomologie 35: 259-266.

[16] Hughes J, Vogler AP (2004) The phylogeny of acorn weevils (genus *Curculio*) from mitochondrial and nuclear DNA sequences: the problem of incomplete data. Mol Phylogenet Evol 32: 601-615. doi:10.1016/j.ympev.2004.02.007. PubMed: 15223041. 15223041 10.1016/j.ympev.2004.02.007

[17] Pélisson PF, Henri H, Bel-Venner MC, Allemand R, Merville A et al. (2011) Identification at the larval stage of four Curculio species coexisting on oak trees using PCR-RFLP. Entomol. Exp Appl 138: 77-82. doi:10.1111/j.1570-7458.2010.01077.x.

[18] Venner S, Pélisson PF, Bel-Venner MC, Débias F, Rajon E et al. (2011) Coexistence of Insect Species Competing for a Pulsed Resource: Toward a Unified Theory of Biodiversity in Fluctuating Environments. PLOS ONE 6(3): e18039. doi:10.1371/journal.pone.0018039. PubMed: 21445318. 21445318 10.1371/journal.pone.0018039 PMC3061935

[19] Kelly D, Sork VL (2002) Mast seeding in perennial plants: Why, How, Where? Annu Rev Ecol Syst 33: 427-447. doi:10.1146/annurev.ecolsys.33.020602.095433.

[20] Sork VL, Bramble J, Sexton O (1993) Ecology of mast-fruiting in three species of North American deciduous oaks. Ecology 74: 528-541. doi:10.2307/1939313.

[21] Williamson M (1966) Premature abscissions and white oak acorn crops. Forest Sci 12: 19-21.

[22] Cecich RA, Sullivan N (1999) Influence of weather at time of pollination on acorn production of *Quercus* *alba* and *Quercus* *velutina* . Can J Forest Res 29: 1817–1823. doi:10.1139/x99-165.

[23] Chesson P, Gebauer RLE, Schwinning S, Huntly N, Wiegand K et al. (2004) Resource pulses, species interactions, and diversity maintenance in arid and semi-arid environments. Oecologia 141: 236-253. PubMed: 15069635. 15069635 10.1007/s00442-004-1551-1

[24] Mathias A, Chesson P (2013) Coexistence and evolutionary dynamics mediated by seasonal environmental variation in annual plant communities. J Theor Biol, 84: 56–71. PubMed: 23287702. 10.1016/j.tpb.2012.11.009 23287702

[25] Pélisson PF, Bel-Venner MC, Rey B, Burgevin L, Martineau F et al. (2012) Contrasted breeding strategies in four sympatric sibling insect species: when a proovigenic and capital breeder copes with a stochastic environment. Funct Ecol 26: 198-206. doi:10.1111/j.1365-2435.2011.01925.x.

[26] Jervis MA, Heimpel GE, Ferns PN, Harvey JA, Kidd NA (2001) Life-history strategies in parasitoid wasps: a comparative analysis of “ovigeny.”. J Anim Ecol 70: 442-458. doi:10.1046/j.1365-2656.2001.00507.x.

[27] Jervis MA, Ellers J, Harvey JA (2008) Resource acquisition, allocation, and utilization in parasitoid reproductive strategies. Annu Rev Entomol 53: 361-385. doi:10.1146/annurev.ento.53.103106.093433. PubMed: 17877453. 17877453 10.1146/annurev.ento.53.103106.093433

[28] Giron D, Casas J (2003) Mothers reduce egg provisioning with age. Ecol Lett 6: 273–277. doi:10.1046/j.1461-0248.2003.00429.x.

[29] Perrin N, Sibly RM (1993) Dynamic models of energy allocation and investment. Annu Rev Ecol Syst 24: 379–410. doi:10.1146/annurev.es.24.110193.002115.

[30] Pélisson PF (2011) Ressources pulsées et coexistence d’espèces en compétition: le cas d’insectes phytophage. PhD dissertation, Université Lyon1- France, p. 260

[31] Bonnet-Masimbert M (1984) Biologie florale et cycle de reproduction de quelques arbres forestiers. Douglas, pin sylvestre, chêne. In Pesson P Louveaux J Pollinisation et productions végétales. France: INRA pp. 219-242.

[32] Wessels FJ, Jordan DC, Hahn DA (2010) Allocation from capital and income sources to reproduction shift from first to second clutch in the flesh fly, *Sarcophaga* *crassipalpis* . J Insect Physiol 56: 1269-1274. doi:10.1016/j.jinsphys.2010.03.033. PubMed: 20417214. 20417214 10.1016/j.jinsphys.2010.03.033

[33] Casas J, Pincebourde S, Mandon N, Vannier F, Poujol R et al. (2005) Lifetime nutrient dynamics reveal simultaneous capital and income breeding in a parasitoid. Ecology 86: 545–554. doi:10.1890/04-0812.

[34] Varpe Ø, Jørgensen C, Tarling GA, Fiksen Ø (2009) The adaptive value of energy storage and capital breeding in seasonal environments. Oikos 118: 363-370. doi:10.1111/j.1600-0706.2008.17036.x.

[35] Thoren LM, Karlsson PS (1998) Effects of supplementary feeding on growth and reproduction of three carnivorous plant species in a subarctic environment. J Ecol 86: 501-510. doi:10.1046/j.1365-2745.1998.00276.x.

[36] Klaassen M, Abraham K, Jefferies R (2006) Factors affecting the site of investment, and the reliance on savings for arctic breeders: the capital-income dichotomy revisited. Ardea. 94: 371-384.

[37] Andersen R, Gaillard JM, Linnell JDC, Duncan P (2000) Factors affecting maternal care in an income breeder, the European roe deer. J Anim Ecol 69: 672-682. doi:10.1046/j.1365-2656.2000.00425.x.

[38] Boyd IL (2000) State-dependent fertility in pinnipeds: contrasting capital and income breeders. Funct Ecol 14: 623-630. doi:10.1046/j.1365-2435.2000.t01-1-00463.x.

[39] Kemp DJ, Alcock J (2003) Lifetime resource utilization, flight physiology, and the evolution of contest competition in territorial insects. Am Nat 162: 290-301. doi:10.1086/376890. PubMed: 12970838. 12970838 10.1086/376890

