# Correction: From Income to Capital Breeding: When Diversified Strategies Sustain Species Coexistence

**DOI:** 10.1371/annotation/1071416f-b6bc-4296-b652-96784fbcd8bc

**Published:** 2014-01-06

**Authors:** Pierre-François Pélisson, Marie-Claude Bel-Venner, David Giron, Frédéric Menu, Samuel Venner

In the subsection "Determination of the global energy budget," of Materials and Methods, the incorrect reference appears in the sentence: "For that purpose, we conducted colorimetric analyses to simultaneously assay on the same individuals the total lipid, total soluble carbohydrate and total soluble protein contents (see detailed method in [26]).

Instead of Reference 26, this sentence should refer to the detailed method in:

Foray V, Pélisson PF, Bel-Venner MC, Desouhant E, Venner S, Menu, et al. (2012) A handbook for uncovering the complete energetic budget in insects: the van Handel’s method (1985) revisited. Physiol. Entomol. 37: 295-302.

An incorrect reference occurs in the next sentence in that section. Only reference [28] should appear. The correct sentence is: "The total energy budget of a female was then computed using the following equivalence: 16 Joules per mg carbohydrates or proteins and 37.65 Joules per mg lipids [28]." 

