# Supplementary material for: From Income to Capital Breeding: When Diversified Strategies Sustain Species Coexistence
Source: PLoS One. 2013 Sep 27;8(9):e76086. doi: 10.1371/journal.pone.0076086 (PMC3785430; doi:10.1371/journal.pone.0076086)

**Figure S1: Total energy budget of the four *Curculio* species.**

Total amount of energy stored in newly-emerged (grey bars) and lived-trapped breeding (white bars) females of the four *Curculio* species. Box-plots: horizontal bold line, median; box, lower and upper quartiles; dashed lines, 95% confidence interval.


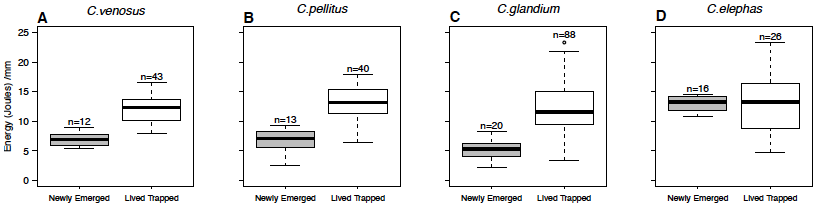

Supplement: Figure S1 — Total energy budget of the four Curculio species. Total amount of energy stored in newly-emerged (grey bars) and lived-trapped breeding (white bars) females of the four Curculio species. Box-plots: horizontal bold line, median; box, lower and upper quartiles; dashed lines, 95% confidence interval. (DOCX) [file pone.0076086.s001.docx]
