# Supplementary material for: From Income to Capital Breeding: When Diversified Strategies Sustain Species Coexistence
Source: PLoS One. 2013 Sep 27;8(9):e76086. doi: 10.1371/journal.pone.0076086 (PMC3785430; doi:10.1371/journal.pone.0076086)

**Figure S2: Time partitioning among the four competing species.**

Representation (in dark) of logistic regression (logit link assuming a binomial distribution of errors) of the ovarian dynamics for each species (presence or absence of eggs in ovaries). The grey lines represent the seasonal dynamic of the whole-body energy budget of females belonging to the four *Curculio* species, presented in the figure 2 and table 1. The comparison of the two models shows the acquisition and allocation dynamics in reproduction.


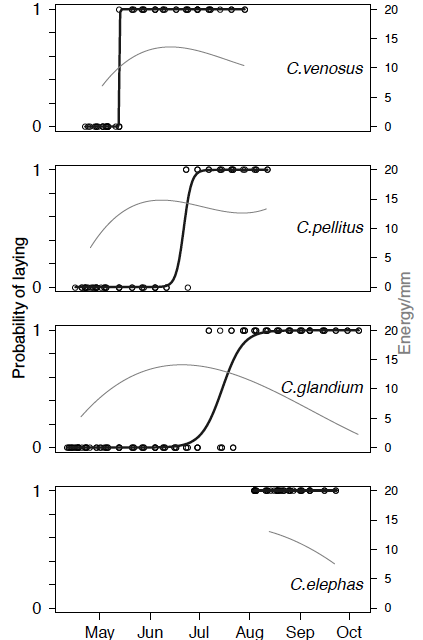

Supplement: Figure S2 — Time partitioning among the four competing species. Representation (in dark) of logistic regression (logit link assuming a binomial distribution of errors) of the ovarian dynamics for each species (presence or absence of eggs in ovaries). The grey lines represent the seasonal dynamic of the whole-body energy budget of females belonging to the four Curculio species, presented in the Figure 1 and table 1. The comparison of the two models shows the acquisition and allocation dynamics in reproduction. (DOCX) [file pone.0076086.s002.docx]
