# Supplementary material for: From Income to Capital Breeding: When Diversified Strategies Sustain Species Coexistence
Source: PLoS One. 2013 Sep 27;8(9):e76086. doi: 10.1371/journal.pone.0076086 (PMC3785430; doi:10.1371/journal.pone.0076086)

**Figure S3: Seasonal nutrient dynamic in females of the four *Curculio* species**

Top (line A) Seasonal dynamic of total lipids for the four species (*C. venosus* A1; *C. pellitus* A2; *C. glandium* A3; *C. elephas* A4). Medium (Line B) Amount of total proteins Low (Line C): Amount of total carbohydrates. Data shown for newly-emerged females (full circles) and lived-trapped females (open circles).


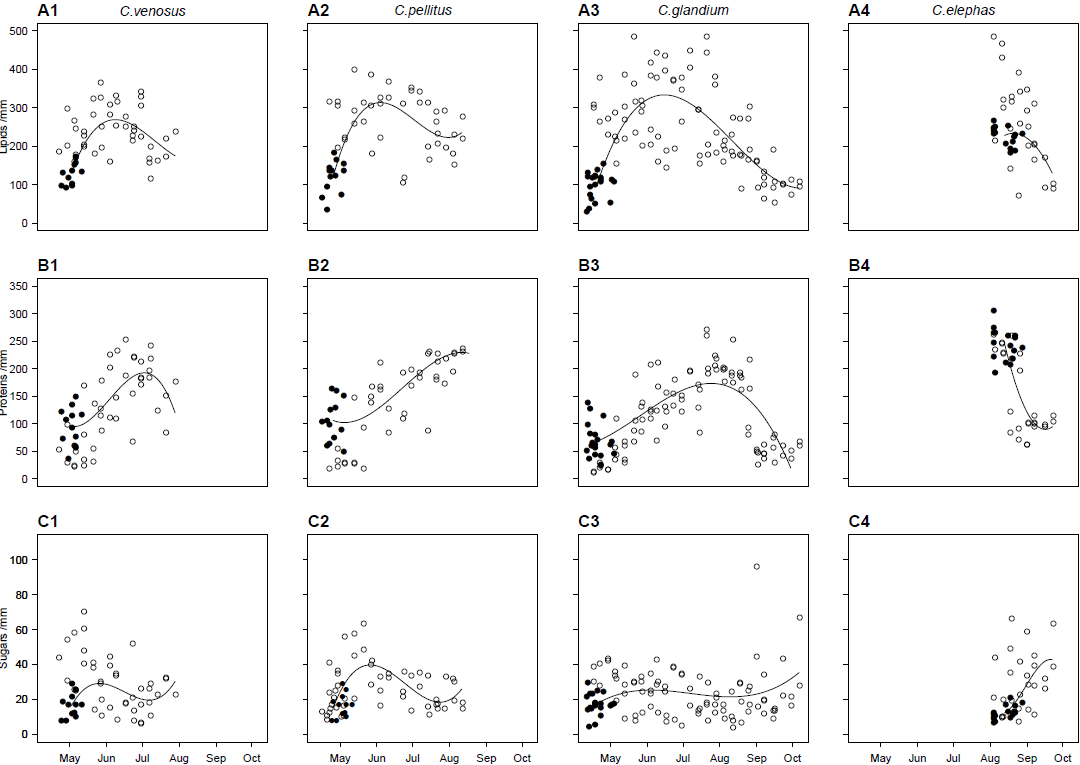

Supplement: Figure S3 — Seasonal nutrient dynamic in females of the four Curculio species. Top (line A) Seasonal dynamic of total lipids for the four species (C. venosus A1; C. pellitus A2; C. glandium A3; C. elephas A4). Medium (Line B) Amount of total proteins Low (Line C): Amount of total carbohydrates. Data shown for newly-emerged females (full circles) and lived-trapped females (open circles). (DOCX) [file pone.0076086.s003.docx]
