# Supplementary material for: From Income to Capital Breeding: When Diversified Strategies Sustain Species Coexistence
Source: PLoS One. 2013 Sep 27;8(9):e76086. doi: 10.1371/journal.pone.0076086 (PMC3785430; doi:10.1371/journal.pone.0076086)

**Table S1: Analyses of the time partitioning between species in the breeding season.**

Logistic regressions of laying probabilities differ between species (interactions species:date). All pairwise tests are significant even by applying a Bonferroni correction for multiple tests.


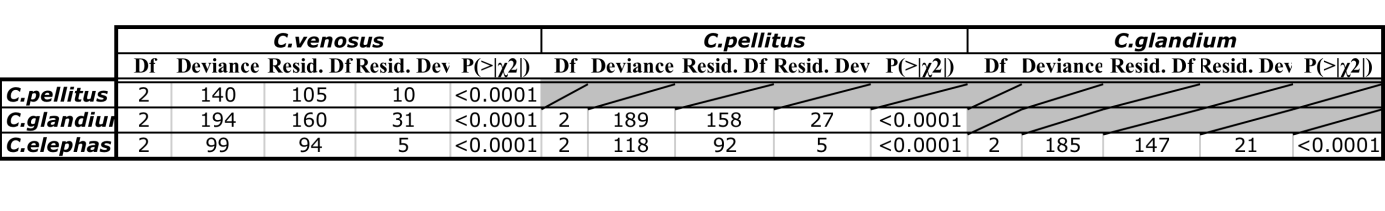

Supplement: Table S1 — Analyses of the time partitioning between species in the breeding season. Logistic regressions of laying probabilities differ between species (interactions species: date). All pairwise tests are significant even by applying a Bonferroni correction for multiple tests. (DOCX) [file pone.0076086.s004.docx]
